# Methodology for the identification of relevant loci for milk traits in dairy cattle, using machine learning algorithms

**DOI:** 10.1016/j.mex.2022.101733

**Published:** 2022-05-16

**Authors:** María Agustina Raschia, Pablo Javier Ríos, Daniel Omar Maizon, Daniel Demitrio, Mario Andrés Poli

**Affiliations:** a Instituto Nacional de Tecnología Agropecuaria, CICVyA-CNIA, Instituto de Genética “Ewald A. Favret”. Hurlingham, Buenos Aires, Argentina; b Universidad de Buenos Aires, Buenos Aires, Argentina; c Instituto Nacional de Tecnología Agropecuaria, E.E.A. Anguil. Anguil, La Pampa, Argentina; d Instituto Nacional de Tecnología Agropecuaria, Dirección General de Sistemas de Información, Comunicación y Procesos - Gerencia de Informática y Gestión de la Información. Buenos Aires, Argentina; e Facultad de Ciencias Exactas, Universidad Nacional de La Plata, Argentina; f Facultad de Agronomía, Universidad Nacional de La Pampa, Argentina; g Facultad de Ciencias Agrarias y Veterinarias, Universidad del Salvador, Argentina

**Keywords:** Single nucleotide polymorphisms, Estimated breeding values, Dairy cattle, XGBoost, LightGBM, Random forest, Milk production, Milk fat content, Milk protein content, EBV_F_, estimated breeding values for milk fat content, EBV_M_, estimated breeding values for milk production, EBV_P_, estimated breeding values for milk protein content, FDR, false discovery rate, GWAS, genome-wide association study, HxJ, Holstein x Jersey, LGB, LightGBM, MAE, mean absolute error, ML, machine learning, MSE, mean squared error, RF, Random Forest, RMSE, root mean square error, SNP, single nucleotide polymorphism, XGB, XGBoost

## Abstract

Machine learning methods were considered efficient in identifying single nucleotide polymorphisms (SNP) underlying a trait of interest. This study aimed to construct predictive models using machine learning algorithms, to identify loci that best explain the variance in milk traits of dairy cattle. Further objectives involved validating the results by comparison with reported relevant regions and retrieving the pathways overrepresented by the genes flanking relevant SNPs. Regression models using XGBoost (XGB), LightGBM (LGB), and Random Forest (RF) algorithms were trained using estimated breeding values for milk production (EBV_M_), milk fat content (EBV_F_) and milk protein content (EBV_P_) as phenotypes and genotypes on 40417 SNPs as predictor variables. To evaluate their efficiency, metrics for actual vs. predicted values were determined in validation folds (XGB and LGB) and out-of-bag data (RF). Less than 4500 relevant SNPs were retrieved for each trait. Among the genes flanking them, signaling and transmembrane transporter activities were overrepresented.

The models trained:

• Predicted breeding values for animals not included in the dataset.
• Were efficient in identifying a subset of SNPs explaining phenotypic variation.

The results obtained using XGB and LGB algorithms agreed with previous results. Therefore, the method proposed could be applied for future association studies on milk traits.

## Graphical abstract

**Figure:**
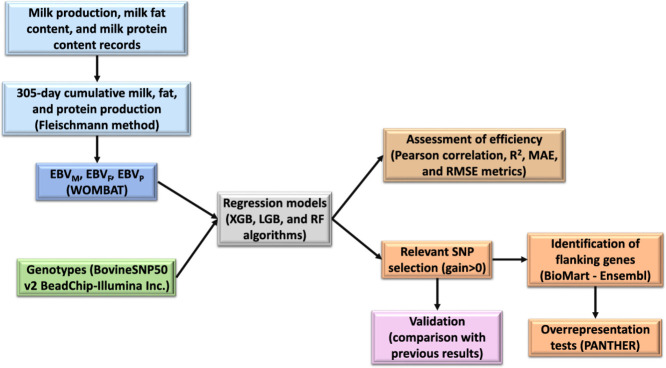

## Specifications table

**Table:** 

Subject Area:	Bioinformatics
More specific subject area:	Machine learning applications in biology
Method name:	Construction of predictive models using machine learning algorithms for the identification of loci that best explain the variance in milk traits of dairy cattle.
Name and reference of original method:	B. Li, N. Zhang, Y.-G. Wang, A.W. George, A. Reverter, Y. Li, Genomic Prediction of Breeding Values Using a Subset of SNPs Identified by Three Machine Learning Methods, Front. Genet. 9 (2018) 237, doi:10.3389/fgene.2018.00237.
Resource availability:	N.A.

## Method details

**Fig. 1:**
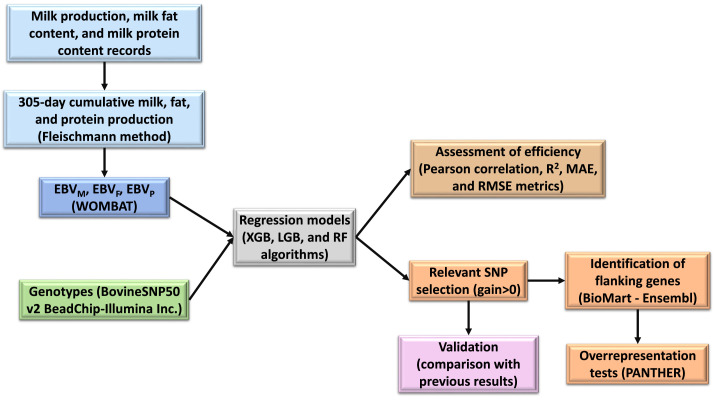
Method workflow. EBV_M_: estimated breeding values for milk production; EBV_F_: estimated breeding values for milk fat content; EBV_P_: estimated breeding values for milk protein content; XGB: XGBoost; LGB: LightGBM; RF: Random Forest; MAE: mean absolute error; RMSE: root mean square error.

In recent years, machine learning (ML) methods have been used in genome-wide association studies (GWAS), showing to be efficient in identifying a subset of SNPs underlying a trait of interest [1,2]. Therefore, we present herein a workflow consisting, firstly, in the construction of predictive models using machine learning algorithms, for the identification of genomic regions that best explain the variance in milk traits of dairy cattle. Once relevant loci are identified, bioinformatics tools and online available software are used to retrieve the genes flanking those loci and explore the metabolic pathways, biological processes and/or molecular functions overrepresented by them. The method workflow is schematically represented in Fig. 1. This method could be suitable to use as an alternative to GWAS performed through the implementation of different software fitting linear, multivariate and Bayesian linear mixed models.

### Phenotypes

A database consisting of 117957 milk production records, 98907 milk fat content records, and 97684 milk protein content records from the first lactation of 18876, 16907, and 16735 Holstein and Holstein x Jersey (HxJ) cows, respectively, was used. Cows had a minimum of four test-day records up to 305 days of lactation, and at least one of these records was taken before 60 days of lactation. Using these productive data, 305-day cumulative milk, fat and protein were estimated by the Fleischmann method [3]. Finally, breeding values for each trait were estimated using the WOMBAT program [4], fitting linear mixed models with fixed effects including the percentage of Holstein background, herd, year of birth, and the combined effect of season and year of first lactation, and the animal genetic random effect distributed proportionally to the relationship matrix as stated by the pedigree.

Three phenotypic datasets were built to train regression models using three ML algorithms XGBoost [5], LightGBM [6], and Random Forest [7] on a population of 863 genotyped animals including 837 cows (582 Holstein and 255 HxJ) and 26 bulls (22 Holstein and 4 Jersey):

(1) estimated breeding values for milk production (EBV_M_);
(2) estimated breeding values for milk fat content (EBV_F_);
(3) estimated breeding values for milk protein content (EBV_P_).

These estimations accounted for the relationship among animals and the phenotypes of genotyped and non-genotyped animals.

### Genotypes

SNP genotyping was performed on 969 cows (703 Holstein and 266 HxJ) and 29 bulls (24 Holstein and 5 Jersey) using the BovineSNP50 v2 BeadChip (Illumina Inc., San Diego, CA, USA), which evaluates 54609 SNP distributed over the 29 bovine autosomes and sex chromosomes, spaced on average 48102 bp apart. The quality control of genotype data was performed using PLINK program v1.07 [8] and consisted in the exclusion of SNP with unknown position on the genome, located on the Y chromosome, with a call rate lower than 0.95 or a minor allele frequency lower than 0.03. Animals with a call rate lower than 0.90 were also excluded. After genomic data quality control, genotypes on 40417 SNP from 978 (952 cows + 26 bulls) animals were retained. However, only 863 out of these animals had reliable phenotypic records. Recoding from A/B to 0/1/2 genotypic notation, in the construction of the input datasets for the models, was also performed with PLINK program v1.07.

### Models trained using machine learning algorithms

Regression models using XGBoost (XGB), LightGBM (LGB), and Random Forest (RF) algorithms were trained using EBV_M_, EBV_F_, and EBV_P_ of 863 animals as phenotypes (target variables) and genotypes on 40417 SNPs as predictor variables. We used Python-based XGB and LGB, and R-based RF algorithms. The final objective of the models was not the prediction of phenotypic values for each animal but the selection or identification of the most important SNPs, i.e. those that best explained the observed variance in the studied phenotypes.

The model hiperparameters adjusted for the three algorithms were the learning rate, max. tree depth, min. number of individuals in leaf nodes, number of features used to create each tree, number of individuals used to create each tree, and regularization values (L1 and L2). These hiperparameters were optimized manually because the relatively small size of the datasets allowed so. The implementation of the algorithms used were XGBoost version 1.3.3 for Python, LightGBM version 3.1.1 for Python, and R package randomForestSRC version 2.10.1. Python release was 3.8.5 and R release was 4.0.4. Source code is available upon request.

The genotypic dataset had a very low amount of missing SNPs, 0.43%, but since Random Forest algorithm discards rows with missing values, it was required to impute them. A multivariate version of missForest imputation was used, which is based on an iterative process using prediction as described by Ishwaran and Kogalur [7].

To evaluate the efficiency of the models in identifying relevant SNPs, Pearson correlation, R^2^, mean absolute error (MAE) and root mean square error (RMSE) metrics for actual vs. predicted values were determined in validation folds using 5-folds cross-validation (XGB and LGB) and out-of-bag data (RF). A 5-fold cross-validation scheme was used based on the study performed by Li et al [9], which used a dataset of comparable size as this study. The models showed, for the three ML algorithms used, R^2^ correlations for actual vs. predicted values greater than 0.34 for EBV_M_, 0.55 for EBV_F_, and 0.57 for EBV_P_, and Pearson correlations, also in validation, greater than 0.61 for EBV_M_, 0.74 for EBV_F_, and 0.76 for EBV_P_. Thus, the models trained learned and were capable to predict breeding values for animals not included in the dataset.

Relevant SNPs retrieved from each trained model were those with importance or gain >0. For Random Forest algorithm, the importance value of a SNP is the percentage of increase in the mean squared error (MSE) in the “out-of-bag” datasets across all the trees in the forest in which the SNP participates, using random permutation. The MSE of each tree in the forest is compared after randomly permuting the values of the variable in a new sample, and the percentage of increase in the error is computed; the larger this error, the more important the variable is. For XGBoost and LightGBM, the importance or gain value of a SNP denotes the reduction in the prediction error of the objective function (MSE) when partitioning a node in a tree using the SNP. The higher the gain value, the more important the SNP.

For EBV_M_, a similar number of relevant SNPs were retrieved out of the 40417 initial evaluated SNPs, with the three ML algorithms used (3633, 4470, and 3718 for XGB, LGB, and RF, respectively). While, both for EBV_F_ and EBV_P_, considerable more SNPs were retrieved when using XGB and LGB than RF algorithms (1774 and 2355 vs. 196 for EBV_F_, and 3040 and 3622 vs. 2493 for EBV_P_, respectively). Common SNPs across the three methods were 514, 133, and 443 for EBV_M_, EBV_F_, and EBV_P_, respectively, representing 14.1, 67.9, and 17.8% of the relevant SNPs detected by the algorithm that retrieved the least number of SNPs with positive importance values for each trait.

### Analysis post relevant SNP identification

Protein-coding genes flanking relevant SNPs in +/- 30 Kb were retrieved by the Ensembl BioMart tool using the bovine genome assembly ARS-UCD1.2 (Ensembl release 104). Then, to assess the biological relevance of the loci identified by each ML algorithm, overrepresentation tests were performed on the gene sets retrieved for each of the nine combinations trait/algorithm, using the program PANTHER (protein annotation through evolutionary relationship) [10]. The parameters used were *Bos taurus* (for organism); statistical overrepresentation test (for analysis method); PANTHER Pathways, PANTHER GO-Slim Biological Process, and PANTHER GO-Slim Molecular Function (for annotation data set); all genes in *Bos taurus* database (for reference list); and Fisher's Exact with FDR multiple test correction (for test type). Results with FDR *p*-value < 0.05 were considered statistically significant.

Protein coding genes containing or flanking relevant SNPs obtained by XGB, LGB, and RF algorithms were 2770, 3334, and 3002 for EBV_M_; 1426, 1889, and 137 for EBV_F_; and 2224, 2804, and 1972 for EBV_P_, respectively. Among those genes and common to the three traits and ML algorithms assayed, signaling pathways and channel activities were detected as overrepresented.

### Method validation

Validation was performed through comparison with previous results. The location of relevant SNPs for EBV_M_, EBV_F_, and EBV_P_ obtained in this study was compared to previously reported relevant 10-adjacent SNP windows that explained more than 10 times genetic variance than expected for milk production, milk fat content, and milk protein content, respectively, obtained using BLUPf90 package of programs for the same population [11]. Then, the number and percentage of relevant windows and top windows (explaining more than 1.0, 0.7, and 0.8% of the genetic variance for milk production, milk fat content, and milk protein content, respectively) containing SNPs with positive gain was determined (Table 1).

**Table 1:** Matching with previous results. The number and percentage of previously reported relevant and top windows for each trait containing SNPs with positive gain obtained in this study is indicated.

Trait	Comparison	XGB	LGB	RF
EBV_M_	relevant windows	40 (76.9%)	46 (88.5%)	40 (76.9%)
	top windows	10 (100%)	10 (100%)	8 (80%)
EBV_F_	relevant windows	33 (57.9%)	33 (57.9%)	3 (5.3%)
	top windows	8 (80%)	6 (60%)	1 (10%)
EBV_P_	relevant windows	44 (78.6%)	47 (83.9%)	27 (48.2%)
	top windows	6 (60%)	9 (90%)	4 (40%)

In general, higher percentages of matching between previous and actual results were obtained with models using XGB and LGB algorithms, than with models using RF. The lower matching reached with RF, mainly evidenced with models trained for EBV_F_ and EBV_P_ traits, can be ascribed to the need to impute missing genotypes when using this algorithm.

Supplementary material *and/or* Additional information:

## Declaration of Competing Interest

The authors declare that they have no known competing financial interests or personal relationships that could have appeared to influence the work reported in this paper.

## References

[1] Leal L.G., David A., Jarvelin M.R., Sebert S., Männikkö M., Karhunen V., Seaby E., Hoggart C., Sternberg M.J.E. (2019). Identification of disease-associated loci using machine learning for genotype and network data integration. Bioinformatics.

[2] Yao C., Spurlock D.M., Armentano L.E., Page Jr C.D., VandeHaar M.J., Bickhart D.M., Weigel K.A. (2013). Random forests approach for identifying additive and epistatic single nucleotide polymorphisms associated with residual feed intake in dairy cattle. J. Dairy Sci..

[3] Craplet C., Thibier M. (1973).

[4] Meyer K. (2007). WOMBAT—a tool for mixed model analyses in quantitative genetics by restricted maximum likelihood (REML). J. Zhejiang Univ. Sci. B.

[5] Chen T., Guestrin C. (2016). Proceedings of the 22nd ACM SIGKDD International Conference on Knowledge Discovery and Data Mining.

[6] Ke G., Meng Q., Finley T., Wang T., Chen W., Ma W., Ye Q., Liu T.Y. (2017). LightGBM: a highly efficient gradient boosting decision tree. Adv. Neural Inf. Process. Syst..

[7] H. Ishwaran, U. Kogalur, Fast unified random forests for survival, regression, and classification (RF-SRC), R package version 2.10.1, https://cran.r-project.org/package=randomForestSRC. (2021)

[8] Purcell S., Neale B., Todd-Brown K., Thomas L., Ferreira M.A.R., Bender D., Maller J., Sklar P., de Bakker P.I.W., Daly M.J., Sham P.C. (2007). PLINK: a tool set for whole-genome association and population-based linkage analyses. Am. J. Hum. Genet..

[9] Li B., Zhang N., Wang Y.-G., George A.W., Reverter A., Li Y. (2018). Genomic prediction of breeding values using a subset of SNPs identified by three machine learning methods. Front. Genet..

[10] Mi H., Muruganujan A., Ebert D., Huang X., Thomas P.D. (2019). PANTHER version 14: more genomes, a new PANTHER GO-slim and improvements in enrichment analysis tools. Nucleic Acids Res..

[11] Raschia M.A., Nani J.P., Carignano H.A., Amadio A.F., Maizon D.O., Poli M.A. (2020). Weighted single-step genome-wide association analyses for milk traits in Holstein and Holstein x Jersey crossbred dairy cattle. Livest. Sci..

